# Consumption of psychotropic drugs among disability pension applicants with mental disorders: comparing awarded and rejected applicants in Finland

**DOI:** 10.1007/s00127-020-01850-8

**Published:** 2020-03-14

**Authors:** Riku Perhoniemi, Annamari Tuulio-Henriksson, Jenni Blomgren, Mikko Laaksonen

**Affiliations:** 1 The Social Insurance Institution of Finland, Nordenskiöldinkatu 12, 00250 Helsinki, Finland; 2 Department of Psychology and Logopedics, University of Helsinki, Helsinki, Finland; 3 Finnish Centre for Pensions, Helsinki, Finland

**Keywords:** Disability pension, Psychotropic drug purchases, Mental health, Trajectories

## Abstract

**Purpose:**

Psychotropic drug consumption as a proxy measure of mental health problems during a disability pension process has only been studied among awarded applicants. This study examined psychotropic drug purchase trajectories among awarded and rejected disability pension applicants. Analyses were conducted in different diagnostic and sociodemographic groups.

**Methods:**

A representative 70% sample of Finnish adults applying for disability pension due to a mental disorder in 2009–2011 (*N* = 18,087) was followed for 4 years in 3-month periods both before and after the pension decision. Register data on purchased drugs measured in defined daily doses (DDDs), gender, age, occupational class, unemployment history, and diagnostic group were used. The DDD levels and trends were analyzed using growth curve models.

**Results:**

Psychotropic drug purchases increased before the pension decision and decreased gradually thereafter among both awarded and rejected applicants. The average DDD level was higher for rejected than awarded applicants before the decision but lower thereafter. The high pre-decision level for rejected applicants was explicit with a lower socioeconomic status. The pre-decision increase in DDDs was steeper for awarded applicants. Changes in DDDs before and after the decision were most prominent for depression, bipolar disorders, schizophrenia, and anxiety disorders.

**Conclusion:**

Awarded and rejected disability pension applicants differed partly in their trajectories of psychotropic drug consumption. For awarded applicants, the steep rise of consumption prior to the award possibly reflects worsening occupational capacity. Early high consumption for rejected applicants signals long running mental health problems and calls for earlier support.

## Introduction

Mental disorders are a major cause for occupational disability worldwide. Worryingly, disability benefits attributable to mental health problems have also been increasing in many OECD countries during the past decades [1, 2]. However, in addition to mental health problems causing disability retirement or exit from the labor market, also disability retirement can affect mental health. A decline in mental health can be a consequence of a loss of meaningful roles in life [3–5]. In addition, the process of applying for disability pension may be a risk for mental health, as it involves decreasing work ability and uncertainty over application outcome. For instance, Øverland et al. [6] found an inversed U-shaped temporal curve in self-reported psychological symptoms among Norwegian disability retirees, with an increase in symptoms around the time of pension award and a decrease thereafter.

Consumption of psychotropic drugs can be considered a proxy measure of mental health status and has been shown to predict future disability pension over and above sociodemographic factors [7–9]. Studies on the trajectories of purchased psychotropic drugs before and after disability retirement in Finland have shown a similar inversed U-shaped curve as described above: there is a notable increase in the amount of purchased drugs during the months preceding the award and a steady decrease thereafter [10–12]. In these studies, the inversed U-shaped curve concerned mostly disability pensions based on mental disorders. The inversed U-curve has not been found among all-cause disability retirees in Norway for psychotropic drug purchases [13] or prescribed medication in general [6]. This lends support to the idea that the increase in medication concerns especially persons receiving disability pension due to a mental disorder. On the other hand, previous studies on psychotropics consumption and disability pension process based on mental disorders are inconclusive. Rahman et al. [14] found only little general temporal variation in antidepressant purchases among Swedish disability retirees with common mental disorders, but instead found multiple trajectory groups.

The literature presented above has only included awarded applicants, thus ignoring the other side of the story. We aim to further understand the association between the disability pension process and the use of psychotropic drugs by including both awarded and rejected applicants. While the financial and labor market challenges for rejected disability pension applicants are clear [15–18], studies on mental health after the pension decision have been mostly qualitative [19, 20]. In one of the rare quantitative studies, Ydreborg et al. [21] found that after the pension decision, those who received a rejection had lower self-reported health, quality of life, and smaller social networks than awarded applicants. The trajectories of psychotropic drug consumption in this group have not been studied. Compared to awarded applicants, rejected applicants often have weaker labor market attachment and lower income, as well as multiple diagnoses with both somatic and psychiatric comorbidity [21–24]. With this background of lesser personal resources, the rejected application itself may impair their mental health.

There is also a clear need for studies comparing psychotropic drug purchases among disability pension applicants with different major mental disorders. Besides the mental disorder diagnosis, sociodemographic factors can also associate with the level and change in psychotropic drug purchases. This should be taken into account when studying drug purchases among persons applying for disability pension.

Based on previous research, our research questions are:

1. What are the levels of psychotropic drug purchases before and after a pension decision for disability pension among awarded and rejected applicants?
2. How does the amount of purchased psychotropic drugs change before and after a disability pension decision among awarded and rejected applicants?
3. How are diagnostic groups of mental disorders and sociodemographic factors associated with the relationship between application outcome (award/rejection) and psychotropic drug purchases?

## Methods

### Disability insurance system and disability pensions in Finland

The Finnish disability pension scheme covers all permanent residents. It includes earnings-related pensions for those with work history and national pensions that secure a minimum pension for those without or with a short work history. Disability pension may be awarded if a person’s work disability is medically assessed to last for at least 1 year. This means that the application is normally preceded by a 1-year period of sickness allowance. Evaluating the applications for disability pension is juridically and medically oriented, with specialists in insurance medicine making the decisions. On average, pension insurers resolve claims in 2 months [25]. Permanent pension is awarded only if rehabilitation is deemed unfeasible. In the last decade, main changes in Finnish disability policies and pensions include a decreasing amount of full-time disability pension applications and a strong rise in the rejection rate, from one-fourth to around one-third [26–28].

Mental disorders are the cause of a significant proportion of disability pensions and a dominant diagnostic group among disability pension applicants [29, 30], accounting for 35% of new applications in 2017 [31].

### Study population

For the years 2009–2011, random register samples comprising 70% of all working-age adult Finnish residents (aged 18–64) were retrieved from the population data file of the Social Insurance Institution of Finland (Kela). The study population, i.e., new applicants for disability pension due to a mental or behavioral disorder in 2009, 2010 or 2011, was formed using registers of the Finnish Centre for Pensions (earnings-related pensions) and the Social Insurance Institution of Finland (national basic-level pensions). An application was considered new if the applicant had no previous disability pension decisions during the 2 years preceding the application. Based on register data from Statistics Finland, those already receiving a pension at the end of the previous calendar year were excluded. Furthermore, only those who had applied for full pension (in contrast to partial pensions) were included. The study sample thus defined consisted of 18,087 individuals.

### Variables

Our data on disability pension decisions included the date which the decision was made by a pension insurer. In this article, we refer to that date when we address the time preceding or following a pension decision (or simply “decision”).

Primary diagnoses of the pension applications were categorized into six groups of mental and behavioral disorders according to the International Statistical Classification of Diseases and Related Health Problems (ICD-10) [32]: (1) substance use disorders (ICD-10: F10–F19), (2) schizophrenia and other psychotic disorders (F20–F29, labeled ‘Schizophrenia’), (3) bipolar disorders and mania (F30–F31, labeled ‘Bipolar’), (4) major depressive disorders (F32–F33, labeled ‘Depression’), (5) anxiety disorders (F40–F48), and (6) other mental disorders. Intellectual disabilities (F70–F79) were excluded.

Register data on drug purchases were retrieved from Kela, and they included defined daily doses (DDD) of purchased psychotropic drugs. Psychotropic drugs included psycholeptics and psychoanaleptics (N05 and N06 in the Anatomical Therapeutic Chemical Classification System) [33] that contain, for instance, antipsychotics, anxiolytics, hypnotics and sedatives, and antidepressants. Anti-dementia drugs were excluded from the study. The amounts of drug purchases were calculated as sums for 3-month periods. Hence, the follow-up was composed of 16 3-month periods 4 years before and similarly 16 periods 4 years after the pension decision. As the calculated DDDs for the 3-month periods in some cases included several drugs, the number of 3-month DDDs could exceed 90.

Covariates of the study were retrieved from the registers of Kela, the Finnish Centre for Pensions and Statistics Finland. Gender and age in four groups were included as demographic factors. Socioeconomic factors included occupational class and unemployment benefit history during the 4 years before applying for disability pension. Occupational class was obtained for the preceding calendar year and followed the classification of Statistics Finland [34]. The group “other” included the long-term unemployed, students, and other persons outside the labor force or without reliable data on occupational class. Unemployment history was coded to unemployment periods covering 0% (none), under 50%, or at least 50% of the four preceding calendar years.

Table 1 shows the distribution of the sociodemographic factors and diagnostic groups (%) among the awarded (*N* = 13,779, 76%) and rejected (*N* = 4308, 24%) disability pension applicants. Chi-square tests (column 1) showed that except for gender, the distributions varied between awarded and rejected applicants. Compared to awarded applicants, rejected applicants were more often 30–49 years old, were more often in the occupational class group “other”, had more often been unemployed during the years before the pension decision, and had less often a bipolar disorder or schizophrenia and more often an anxiety disorder or a substance use disorder as the primary diagnosis. Depression was the most common primary diagnostic group among both awarded and rejected applicants.

**Table 1:** Distribution of the sociodemographic and diagnostic groups (%) among the awarded and rejected disability pension applicants

	Awarded applicants	Rejected applicants
	*N* = 13,779	*N* = 4308
Gender (*p* = 0.051)		
Male	47.7	49.4
Female	52.3	50.6
Age group (*p* < 0.001)		
18–29 years	21.5	15.9
30–39 years	15.9	18.3
40–49 years	22.4	28.8
50–64 years	40.3	37.0
Occupational class (*p* < 0.001)		
Upper non-manual employee	8.1	5.1
Lower non-manual employee	18.2	14.0
Manual worker	16.6	14.4
Entrepreneur	5.0	4.0
Other	52.1	62.4
Unemployment during previous 4 years (*p* < 0.001)		
None	48.0	28.8
Under 50% of 4 years	32.3	41.8
At least 50% of 4 years	19.7	29.4
Primary diagnosis (*p* < 0.001)		
Depression	51.3	55.3
Bipolar disorders	12.0	5.0
Schizophrenia	18.6	1.7
Anxiety disorders	7.0	16.9
Substance use disorders	3.7	11.2
Other mental disorders	7.5	9.9
All	100.0	100.0

### Statistical methods

We analyzed the average levels and trends of the purchased psychotropic drugs with three-month intervals using growth curve modeling [35]. Models were done separately for the 16 3-month periods before and 16 3-month periods after the date of the pension decision. To specify, for the level of drug purchases, the average differences between awarded and rejected applicants were calculated comparing the means of the 3-month periods’ sums. A positive value means that the rejected applicants purchased more psychotropic drugs on average than the awarded applicants. In the analysis of trends, the estimated values represent the mean change between average purchases of the 16 consecutive 3-month periods.

Models were first stratified for each sociodemographic and diagnostic group. As we wanted to account for possible changes in drug purchases due to aging and variation between calendar years, we adjusted for time-varying age and the year of the pension decision. Models were also conducted as unadjusted and adjusted for all covariates (full adjusted) to evaluate covariate effects. As the models divided follow-up time into two phases without considering temporal variation within those phases, visual figures of drug purchases were presented to explore both levels and trends more precisely. This was also done separately for the diagnostic groups.

In addition, since an eventual pension award may affect the results for the initially rejected applicants, a post hoc analysis of drug purchases was conducted for those with only a rejected application and no later award.

The analyses were conducted using Stata statistical software package 14.0.

## Results

**Fig. 1:**
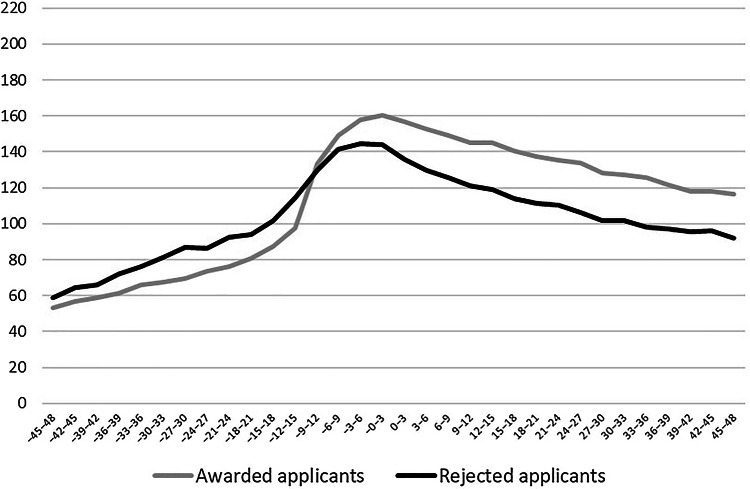
Purchases of psychotropic drugs (defined daily doses, DDD) in 3-month periods before and after applying for disability pension by application outcome

Figure 1 shows the trajectories of purchased psychotropic drugs for awarded and rejected applicants over the 8-year observation period. Among both groups, drug purchases were most common around the date of the pension decision. The figure also shows that before the decision, the level of purchases was slightly higher for the rejected than for the awarded applicants. However, approximately 18–12 months before the decision, the drug purchases of those who were eventually awarded pension steeply increased and caught up with the average drug purchase level of rejected applicants and exceeded it when the pension decision was made. Following this increase, the awarded applicants had a higher purchase level than rejected applicants during the 4 years after the decision.

### The average level of psychotropic drug purchases

**Fig. 2:**
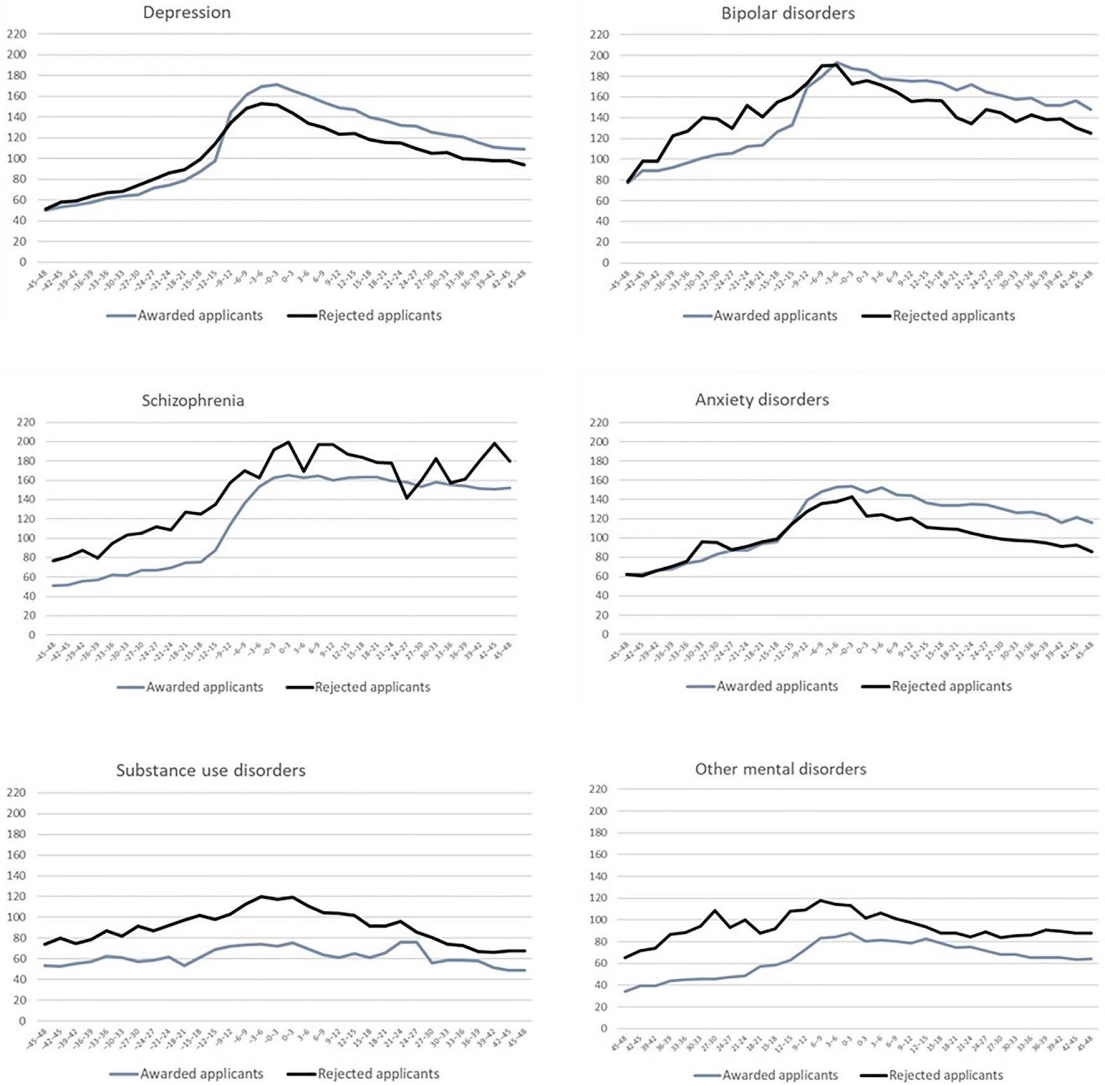
Purchases of psychotropic drugs (defined daily doses, DDD) in 3-month periods before and after applying for disability pension by application outcome and diagnostic group

Average differences in the levels of drug purchases between rejected and awarded applicants by sociodemographic and diagnostic groups are presented in Table 2. During the 4 years before the pension decision, the amount of purchased drugs during each 3-month period was, on average, 10.6 doses higher for rejected applicants than for awarded applicants when time-varying age and calendar year were adjusted for. The difference attenuated slightly in the fully adjusted model (9.7 DDDs). Table 2 also presents the average differences in purchased psychotropic drugs between awarded and rejected applicants by covariate subgroups (adjusted for time-varying age and calendar year). Rejected applicants had a clearly and statistically significantly higher average amount of purchased psychotropic drugs than the awarded applicants in the following groups: men, the youngest and oldest age groups, those in the occupational class ‘other’, and those with either no previous unemployment or who were unemployed for at least 50% of the four preceding years. The difference between rejected and awarded applicants was highest in the diagnostic groups of bipolar disorders, schizophrenia, and other mental disorders. Drug purchases by diagnostic groups are also shown in Fig. 2. As can be seen, the level of drug purchases for rejected applicants is consistently higher also among those applying for pension due to substance use disorders, but the difference was not statistically significant, as shown in Table 2.

**Table 2:** Average level of purchased psychotropic drugs among rejected applicants as compared to awarded applicants before and after applying for disability pension by the study variables (95% confidence intervals)

	Before application		After application	
		CI		CI
All—unadjusted	15.4	10.8 to 19.9	− 18.8	− 24.8 to − 12.8
All—adjusted for age, year	10.6	6.0 to 15.2	− 23.2	− 29.2 to − 17.3
All—full adjusted	9.7	4.9 to 14.4	− 17.6	− 23.7 to − 11.5
By covariates				
Gender				
Male	15.3	8.2 to 22.5	− 18.1	− 27.3 to − 8.8
Female	6.1	0.4 to 11.8	− 28.5	− 36.3 to − 20.8
Age group				
18–29 years	16.6	9.0 to 24.3	− 17.7	− 31.8 to − 3.6
30–39 years	14.7	0.4 to 29.0	− 16.8	− 32.8 to − 0.8
40–49 years	7.1	− 3.0 to 17.1	− 38.6	− 51.2 to − 26.1
50–64 years	14.4	8.0 to 20.7	− 12.2	− 20.7 to − 3.7
Occupational class				
Upper non-manual employee	3.6	− 14.7 to 21.9	− 40.4	− 63.3 to − 17.6
Lower non-manual employee	− 6.8	− 16.0 to 2.3	− 39.8	− 53.3 to − 26.3
Manual worker	8.1	− 3.0 to 19.2	− 34.7	− 49.7 to − 19.9
Entrepreneur	13.9	− 9.0 to 36.7	− 16.1	− 43.0 to 10.8
Other	13.8	7.4 to 20.1	− 12.7	− 20.9 to − 4.4
Unemployment during previous 4 years				
None	14.5	7.6 to 21.4	− 24.7	− 34.6 to − 14.8
Under 50% of 4 years	2.4	− 4.7 to 9.5	− 28.1	− 37.4 to − 18.8
At least 50% of 4 years	11.6	0.7 to 22.5	− 9.0	− 22.2 to 4.3
Primary diagnosis				
Depression	8.4	3.0 to 13.8	− 26.5	− 34.3 to − 18.7
Bipolar disorders	28.1	8.0 to 48.3	− 5.1	− 28.1 to 18.0
Schizophrenia	28.1	0.4 to 55.8	8.3	− 32.7 to 49.4
Anxiety disorders	− 3.1	− 19.5 to 13.3	− 21.1	− 38.1 to − 4.1
Substance use disorders	8.6	− 13.6 to 30.8	9.6	− 16.5 to 35.7
Other mental disorders	25.6	9.6 to 41.7	6.0	− 13.8 to 25.9

Average difference in defined daily doses (DDD) in the 3-month periods, rejected minus awarded. Adjusted for applicant age and calendar year of the application where not notified otherwise

After the pension decision, the average amount of purchased psychotropic drugs was 23.2 doses lower for rejected applicants than for awarded applicants (adjusted for age and calendar year). The average difference somewhat attenuated with adjusting for all covariates (− 17.6 DDDs). The average amount of purchased psychotropic drugs was statistically significantly lower for rejected applicants in almost all sociodemographic groups and among those with depression or an anxiety disorder. Figure 2 also shows the difference for these two diagnostic groups. Furthermore, the figure shows a lower drug purchase level among rejected applicants than among awarded applicants in the group of bipolar disorders, but the difference was not statistically significant. In contrast, among those who applied due to schizophrenia, substance use disorders, and other mental disorders, the drug purchase level was consistently higher for the rejected than for the awarded applicants also over the 4-year period after the decision. The difference was, however, not statistically significant in these three diagnostic groups.

### Average change in psychotropic drug purchases

During the 4 years before the pension decision*,* purchases of psychotropic drugs increased slightly more strongly among awarded applicants (6.9 DDDs per each 3-month period) than among rejected applicants (6.3 DDDs) when time-varying age and calendar year were adjusted for (Table 3). A statistically significant increase was found in every subgroup of covariates among both awarded and rejected applicants. However, judging from non-overlapping confidence intervals, the increase was statistically significantly steeper among the awarded applicants only among women, applicants at least 40 years old, upper non-manual employees, and in the diagnostic group of depression.

**Table 3:** Average change in purchases of psychotropic drugs before and after applying for disability pension by application outcome and study variables (average and 95% confidence intervals)

	Before application				After application			
	Awarded applicants		Rejected applicants		Awarded applicants		Rejected applicants	
		CI		CI		CI		CI
All—unadjusted	7.0	6.6 to 7.2	6.1	5.7 to 6.4	− 2.7	− 2.9 to − 2.5	− 2.7	− 3.0 to − 2.3
All—adjusted for age, year	6.9	6.8 to 7.1	6.3	5.9 to 6.6	− 2.4	− 2.7 to − 2.2	− 2.6	− 2.9 to − 2.2
All—full adjusted	6.9	6.7 to 7.1	6.2	5.9 to 6.6	− 2.4	− 2.6 to − 2.2	− 2.6	− 2.9 to − 2.2
By covariates								
Gender								
Male	6.8	6.5 to 7.0	6.2	5.7 to 6.8	− 2.4	− 2.8 to − 2.1	− 2.6	− 3.2 to − 2.0
Female	7.1	6.9 to 7.4	6.2	5.8 to 6.7	− 2.4	− 2.7 to − 2.1	− 2.5	− 3.0 to − 2.0
Age group								
18–29 years	7.8	7.5 to 8.2	8.0	7.2 to 8.9	− 1.5	− 2.0 to − 0.9	− 2.2	− 3.1 to − 1.2
30–39 years	7.8	7.3 to 8.3	7.5	6.5 to 8.4	− 2.2	− 2.8 to − 1.7	− 3.0	− 4.1 to − 1.9
40–49 years	7.2	6.8 to 7.6	5.9	5.3 to 6.5	− 3.1	− 3.6 to − 2.6	− 2.5	− 3.2 to − 1.9
50–64 years	6.2	6.0 to 6.4	4.7	4.3 to 5.1	− 3.3	− 3.6 to − 3.1	− 2.8	− 3.2 to − 2.3
Occupational class								
Upper non-manual employee	8.6	7.9 to 9.4	6.4	5.2 to 7.6	− 3.0	− 3.8 to − 2.1	− 2.5	− 4.0 to − 1.0
Lower non-manual employee	8.0	7.6 to 8.4	8.0	7.2 to 8.7	− 3.1	− 3.6 to − 2.6	− 2.7	− 3.6 to − 1.8
Manual worker	8.0	7.5 to 8.5	7.3	6.2 to 8.4	− 2.3	− 2.8 to − 1.8	− 2.3	− 3.1 to − 1.5
Entrepreneur	6.8	6.0 to 7.5	5.9	4.2 to 7.6	− 2.8	− 3.7 to − 1.9	− 4.6	− 6.3 to − 3.0
Other	6.1	5.8 to 6.3	5.5	5.0 to 5.9	− 2.1	− 2.4 to − 1.8	− 2.5	− 3.0 to − 1.9
Unemployment during previous 4 years								
None	7.6	7.3 to 7.9	6.9	6.2 to 7.5	− 2.7	− 3.1 to − 2.4	− 3.2	− 3.9 to − 2.5
Under 50% of 4 years	7.1	6.8 to 7.5	6.8	6.3 to 7.3	− 2.0	− 2.4 to − 1.6	− 2.2	− 2.7 to − 1.6
At least 50% of 4 years	5.4	4.9 to 5.9	4.9	4.2 to 5.6	− 2.5	− 3.0 to − 2.1	− 2.4	− 3.1 to − 1.6
Primary diagnosis								
Depression	8.1	7.9 to 8.4	7.2	6.7 to 7.6	− 3.3	− 3.6 to − 3.0	− 3.0	− 3.5 to − 2.5
Bipolar disorders	6.5	5.6 to 7.3	6.4	4.7 to 8.0	− 2.4	− 3.1 to − 1.7	− 4.2	− 5.9 to − 2.4
Schizophrenia	6.5	6.1 to 7.0	6.6	3.2 to 10.1	− 0.7	− 1.2 to − 0.1	2.3	− 2.1 to 6.7
Anxiety disorders	6.3	5.5 to 7.1	5.8	4.9 to 6.8	− 1.8	− 2.6 to − 1.0	− 2.7	− 3.6 to − 1.9
Substance use disorders	2.1	0.9 to 3.3	4.3	2.8 to 5.8	− 1.6	− 2.6 to − 0.6	− 1.5	− 3.1 to 0.2
Other mental disorders	3.1	2.4 to 3.7	3.1	2.0 to 4.3	− 1.1	− 1.8 to − 0.4	− 0.6	− 1.8 to 0.6

Average change in defined daily doses in consecutive 3-month periods. Adjusted for applicant age and calendar year of the application where not notified otherwise

The increase in psychotropic drug purchases before the decision was also evident in almost all diagnostic groups in Fig. 2 but most prominent among the awarded applicants in the diagnostic groups of depression, bipolar disorders, schizophrenia, and anxiety disorders. Drug purchases increased steeply in these diagnostic groups, especially 18–12 months before the pension was awarded.

After the pension decision, the steepness of the decrease in drug purchases was similar among awarded and rejected applicants. A statistically significant decrease was found in almost every subgroup of covariates. However, the difference between awarded and rejected applicants in the steepness of the decrease was not statistically significant in any subgroup.

Over one-fourth (27%) of the initially rejected applicants were awarded disability pension during the following 4 years. To examine whether the drug purchase trends reflected this especially after an initial rejection, a growth curve analysis was conducted separately for those with only a rejected application and no later award (*N* = 3143). The analysis (available upon request) showed a very similar trajectory to the less strictly defined group of rejected applicants.

## Discussion

Previous studies have shown a link between the disability pension process and the amount of psychotropic drug purchases among pension recipients. Particularly, a considerable rise has been detected in purchases prior to pension awards. The rise applies especially to disability retirement due to mental disorders [10–12]. In the present study, we aimed to further understand this association between the disability pension process and psychotropic drug consumption as a proxy measure of mental health by examining not only awarded but also rejected applicants. As there is a lack of studies comparing psychotropic drug purchases between major mental disorders, we conducted our analyses in different diagnostic groups.

Our results show that among both awarded and rejected disability pension applicants, psychotropic drug purchases increase during the 4 years preceding the pension decision. Drug purchases are most common around the date of the decision. During the next 4 years, purchases of psychotropic drugs decrease gradually. These trends can be a consequence of several simultaneous factors. Based on the difference in trajectories between disability and old-age retirees, Leinonen et al. [11] concluded that the increase in psychotropic drug purchases does not mainly reflect the stressfulness of the pension process, but rather the worsening symptoms associated with occurring disability. Similarly, the decline in purchases may reflect an absence of work-related strains or fewer attempts to restore work ability. In deed, Halonen et al. [36] have shown that for mental health-based disability retirees, the pre-retirement level of psychotropic drug purchases can depend on perceived pre-retirement work stress, while this effect attenuates after retirement. Laaksonen et al. [10] respectively assume that the increase in psychotropic drug consumption among disability retirees reflects the fact that health care and pension systems identify those with disabling health problems.

We are cautious in our interpretation, since the inversed U-shaped curve was found for awarded and rejected applicants alike. The rise in drug purchases probably mainly reflects a worsening mental disorder for other reasons than the stressful application process itself, and especially so for those who are awarded pension. Still, the stressfulness of the pension process may be an additional contributor: big personal transformations in labor market circumstances tend to increase the consumption of psychotropic drugs [37, 38]. The same may apply here if drug consumption is used as a coping mechanism during an uncertain period [39]. Furthermore, an equivalent pre-pension rise and gradual decrease have been found in psychological symptoms around all-cause disability pension awards [6]. The peak of drug consumption around 6–0 months before the decision may show a final stress peak when waiting for the decision. Another valid reason for the inverse U-shaped trajectory is the increasing medical treatment as sickness allowance and application routines with an evaluation of remaining occupational ability entail frequent medical contacts.

Despite the similarities in drug consumption trajectories between awarded and rejected applicants, there may be several underlying causes for the inversed U-curve trajectory. Furthermore, our results show different trajectories for awarded and rejected applicants that need to be discussed.

### Awarded and rejected applicants before the pension decision

For awarded applicants, the initially lower level of psychotropic drugs and the steep rise of drug purchases right before the pension award indicate that the health care and pension systems successfully identify those whose condition severely worsens [10]. The steep rise in purchased psychotropic drugs starts 18–12 months prior to the pension award. This is approximately the timepoint when the sickness allowance period starts for the majority that eventually uses the maximum allowance period of about 1 year and then applies for disability pension. The steeper increase in drug purchases for awarded applicants may then additionally reflect the fact that especially the awarded applicants are known to go through the year-long sickness allowance period [29], therefore coming under intensive medical care. Policies in many countries increasingly prioritize rehabilitation over disability benefits, and a rehabilitation period is usually a precondition for an awarded disability pension [40].

Our study is the first to examine psychotropic drug consumption trajectories among rejected disability pension applicants. Compared to awarded applicants, they are characterized by a higher average psychotropic drug consumption level several years before the pension decision. Rejected applicants are, in general, known to have weaker labor market attachment, a lower level of education and income, and multiple and complex psychiatric diagnoses more often than awarded applicants [16, 22–24]. In turn, those with lower income have less accessible and available health services in Finland [41], and are without the realm of occupational health services. Thus, they may receive less preventive, good-quality psychological treatment. Instead, lower socioeconomic status is known to associate with psychotropic drug treatment [42] and polypharmacy [43]. Adjusting for the socioeconomic covariates did not remove the difference in drug consumption levels, but the difference between awarded and rejected applicants was most apparent with the lowest socioeconomic groups.

Psychiatric comorbidity can also partly account for a higher general drug purchase level of these applicants. Not only internationally but also in Finland, rejected disability pension applicants have more diagnoses on average than awarded applicants [28]. In addition, among Finns who have mental disorders, lower socioeconomic status and higher comorbidity have been shown to associate with higher odds of treating major depressive disorders and anxiety disorder with several psychotropic drugs [43, 44].

In highlight, these results indicate successful identification of worsening occupational capacity for those eventually awarded pension, but also long-standing mental health problems among rejected applicants, calling for earlier support.

### Awarded and rejected applicants after the pension decision

For many, the decreasing amount of drug consumption probably reflects improving mental health. For awarded applicants, this may be due to reduced stress and absence of work-related strains [36, 45]. For some of the awarded applicants, lowering drug purchase levels could in contrast reflect a decreasing incentive to improve functional capacity once the pension benefit is reached. Respectively, for a significant part of the rejected applicants, decreasing drug purchases probably express diminishing symptoms. Specialist physicians evaluating occupational capacity when a pension application is assessed may perceive potential for improvement in work ability and thus reject the application. This anticipated improvement in health may then also demonstrate as decreasing drug consumption. Regardless of application outcome, the decrease in drug consumption can also reflect successful rehabilitation, after which medication levels may be lowered.

However, rather than improving health, for some, the decrease in psychotropic drug consumption may also reflect passivity in the care of persons left without attention from professionals. Being outside social networks, active health professionals’ contact, community, or even society can passivate both those who have been awarded pension and especially those whose application has been rejected [22]. Previous studies from Finland showing a high frequency of unemployment after a rejected disability pension [16, 46] suggest this possibility. Our study cannot confirm the contrary phenomena of improved health or passivity in care.

Although the amount of drug purchases decreased gradually after the pension decision in both application groups, the average level remained higher for the awarded applicants. In addition to reflecting actual disability identified by health care and pension systems, the higher level of psychotropic drug purchases among awarded applicants may also be partly explained by the medication-only treatment more characteristic of pensioners than other patient groups [44].

In general, it is understandable that for both awarded and rejected applicants even 4 years after applying for pension, the drug purchase level remained higher than 4 years before the decision. For the awarded applicants, in addition to the worsening of mental health leading to retirement, disability pension as an involuntary retirement is associated with emerged psychological symptoms [47–49]. A rejected pension may also create long-lasting stress [19, 22], especially with additional financial challenges [15–18].

### Variation in diagnostic and sociodemographic groups

Even though the higher level of drug consumption before the decision among rejected applicants was not fully explained by diagnostic groups, the difference was emphasized among persons suffering from bipolar disorders, schizophrenia, or other mental disorders requiring constant drug treatment [50]. Frequent psychiatric comorbidity in bipolar disorders and schizophrenia with other mental disorders [51, 52] may account for some of this effect.

After the pension decision, the psychotropic drug purchase level was higher for awarded applicants than rejected applicants, especially in the groups with major depressive and anxiety disorders. These disorder groups include a broad range of conditions with very different detriment on occupational ability (e.g., from mild to severe depression, from specific to generalized anxiety), and the severity of depression and anxiety predicts long-term disability [53–55]. Thus, if the health and pension systems are functional in demarcating the disabled from those with a less disabling condition, the broad range of symptom severity within depression and anxiety disorders may result in a clear difference between the awarded and rejected applicants in psychotropic drug consumption.

The higher drug purchase level of rejected applicants before the pension decision depended on occupational class. When stratified, the effect was found only among the group “other”, which includes the long-term unemployed, students, and other persons outside the labor force. This again highlights the associations mentioned above between lower socioeconomic status and general ill-health among rejected applicants. After the decision, there was no equivalent statistically significant effect for occupational class, although the lower drug purchase level of the rejected applicants was least explicit among the group “other”.

## Strengths and limitations

This study is the first to follow psychotropic drug purchases for both awarded and rejected applicants around the time of disability pension decision. The data used were representative of the Finnish working-age population. Data obtained from national registers are considered to be highly reliable, and have objective measures and very little missing information.

However, there are some limitations that warrant caution with the results. The DDD as a statistical unit of measurement does not conclusively reflect the prescribed doses or the actual disorder for which the drugs were prescribed. With only primary diagnoses in data, we were also unable to control for possible psychiatric comorbidity. However, the DDD is an internationally accepted unit in drug consumption studies and the best available measure for this study. In addition, summing DDDs of multiple psychotropic drugs may hide differences in their typical usage (regular versus occasional). The 3-month intervals in our study partly compensate for this possible temporal variation. Furthermore, a sensitivity analysis using a dichotomous measure for purchases (yes/no) validated our main results.

More generally, psychotropic drug purchases are an indirect indicator of mental health status, and we lacked information on actual symptoms. However, register-based data allowed a large sample, which is usually difficult to reach with clinical data collection. We have considered different interpretations for the results and concluded that, depending on the circumstances, changes in drug consumption may reflect several positive or negative changes in pension applicants’ mental health. Future studies should include longitudinal data on more direct indicators of mental health and capability, such as symptoms and rehabilitation outcomes, to strengthen the interpretations based on psychotropic drug consumption.

## Conclusion

For awarded disability pension applicants, the steep rise of psychotropic drug consumption prior to the award possibly reflects the identification of a substantive worsening occupational capacity and better treatment. Respectively, a higher average drug purchase level during the years before the pension decision among rejected applicants indicates lower overall well-being.

Since there was an increase in psychotropic drug consumption starting at least 4 years prior to the pension decision, preventive treatment and targeting well-timed intervention actions could prevent the aggravation of mental disorders. This could decrease early permanent disability pensions, allow persons with mental health problems to remain active, as well as diminish public spending on disability. Active policies would also place disability pension applicants in a more equal position at the time of the pension application and possibly reduce the amount of rejected applications.
